# Genome-Wide Identification and Characterization of the Trehalose-6-phosphate Synthetase (TPS) Gene Family in Watermelon (*Citrullus lanatus*) and Their Transcriptional Responses to Salt Stress

**DOI:** 10.3390/ijms23010276

**Published:** 2021-12-28

**Authors:** Gaopeng Yuan, Junpu Liu, Guolin An, Weihua Li, Wenjing Si, Dexi Sun, Yingchun Zhu

**Affiliations:** Zhengzhou Fruit Research Institute of the Chinese Academy of Agricultural Sciences, Zhengzhou 450000, China; yuangaopeng@caas.cn (G.Y.); liujunpu@caas.cn (J.L.); anguolin@caas.cn (G.A.); liweihua@caas.cn (W.L.); siwenjingsmile@sina.cn (W.S.)

**Keywords:** gene family, watermelon, trehalose-6-phosphate synthetase, salt stress

## Abstract

With the increase in watermelon cultivation area, there is an urgent need to explore enzymatic and genetic resources for the sustainable development of watermelon, especially under salt stress. Among the various compounds known, trehalose plays an important role in regulating abiotic stress tolerances in diverse organisms, including plants. Therefore, the present study comprehensively analyzed the trehalose-6-phosphate synthase (*TPS*) gene family in watermelon. The study analyzed the functional classification, evolutionary characteristics, and expression patterns of the watermelon *TPS* genes family. Seven *ClTPSs* were identified and classified into two distinct classes according to gene structure and phylogeny. Evolutionary analysis suggested the role of purifying selection in the evolution of the *TPS* family members. Further, *cis-*acting elements related to plant hormones and abiotic stress were identified in the promoter region of the *TPS* genes. The tissue-specific expression analysis showed that *ClTPS* genes were widely expressed in roots, stems, leaves, flowers, and fruits, while *ClTPS3* was significantly induced under salt stress. The overexpression of *ClTPS3* in *Arabidopsis thaliana* significantly improved salt tolerance. Finally, the STRING functional protein association networks suggested that the transcription factor ClMYB and ClbHLH regulate *ClTPS3*. Thus, the study indicates the critical role of *ClTPS3* in watermelon response to salt stress.

## 1. Introduction

Trehalose (α-d-glucopyranosyl-1, 1-α-d-glucopyranoside) is a non-reducing disaccharide consisting of two glucose molecules linked by α, α, 1, 1-glycosidic bonds [1]. It has a symmetrical structure, with two glucose molecules having symmetry more stable than maltose, sucrose, glucose, and other sugars of small molecules [2,3]. Therefore, the physical and chemical properties of trehalose are different from its analogs, making it an important component in keeping cells alive. Trehalose is known as a living substance, which exists in all living organisms and plays a major role in plant growth and development [4,5]. In recent years, trehalose has attracted extensive attention as a potential signal metabolite and a cell stabilizer of plants. Cells exposed to high temperature, freezing temperature, radiation, drought, high osmotic pressure, high salinity, and other adverse environmental conditions synthesize trehalose in large quantities, which plays an important role in maintaining osmotic pressure, protecting membrane structure, and participating in the signal transduction process. Subsequently, once the crisis is resolved, trehalose decomposes rapidly to substances that act as energy sources [2].

Trehalose in plants was first identified in *Selaginella lepidophylla* (Hook. and Grev.) Spring almost 100 years ago. A previous study found that high trehalose levels in *Selaginella* helped it to survive under an extreme drought environment [3]. However, in *Arabidopsis thaliana* and other drought-resistant species, only a small amount of trehalose was detected despite the existence of multiple genes encoding trehalose, which may be related to the co-involvement of its precursor trehalose-6-phosphate (T6P) in the regulation of plant stress [6,7]. These earlier findings suggested that trehalose metabolism regulates the biotic and abiotic stress response and may be an important target for improving the stress tolerance of plants.

The T6P signaling pathway may directly regulate many physiological plant activities, such as seed germination, seedling growth, flowering, and senescence [8,9,10]. In plants, T6P is mainly present in the cytoplasm and also in vacuoles and chloroplasts in small amounts. During metabolism, uridine diphosphate glucose (UDPG) and 6-phosphate glucose (G6P) are catalyzed by trehalose-6-phosphate synthetase (TPS) to T6P, and T6P is further catalyzed by trehalose-6-phosphate phosphatase (TPP) to trehalose. Finally, trehalase (TRE) catalyzes the conversion of trehalose to two glucose molecules [11]. In the above metabolic pathway, the TPS protein encoded by the *TPS* gene is the synthetase that catalyzes T6P. *TPS* genes have been identified in different plants, for example, there are eleven *TPS* members in *A. thaliana*, eleven in rice, twelve in poplar, eight in potato, fourteen in rubber tree, twelve in winter wheat, thirteen in apple, twelve in corn, nine in sugarcane, seven each in melon and cucumber, and twenty in soybean [12,13].

Generally, plants have low trehalose content; however, overexpression of the *TPS* gene will increase the trehalose content and improve stress tolerance [14,15,16]. Transgenic plants overexpressing the *TPS* gene improved water-holding power and electrolyte leakage under drought stress [17]. Guo et al. found that the transgenic tobacco line overexpressing *AtTPS* showed a higher trehalose level and enhanced salt tolerance [18]. The overexpression of *HbTPS1* from Pará rubber tree in *A. thaliana* improved tolerance to freezing, heat, and drought stresses [19]. The drought-resistant transgenic plants obtained by homologous transformation of maize *ZmTPS* showed better drought tolerance than the control plants under greenhouse conditions [20]. Meanwhile, exogenous trehalose avoided biofilm damage under extreme conditions, such as low temperature, water loss, hyperosmotic stress, and nutritional imbalance [21,22]. In addition, exogenous trehalose increased the chlorophyll content of flue-cured tobacco seedlings under a low nitrogen environment [23,24], significantly alleviated leaf wilting caused by low temperature, increased antioxidant enzyme activities, reduced membrane lipid peroxidation caused by low temperature, increased leaf water content, and promoted osmotic regulatory substances accumulation [25,26]. Meanwhile, trehalose regulated the root physiological level, improved tolerance, and promoted root growth and biomass increase in maize seedlings under low-temperature stress [27]. In addition, exogenous trehalose pretreatment significantly alleviated the growth state of watermelon cells under mannitol osmotic stress [28]. The application of exogenous trehalose enhanced the drought tolerance and alleviated the drought damage; trehalose upregulated the activities of antioxidant enzymes, such as superoxide dismutase (SOD), ascorbic acid peroxidase (APX), peroxidase (POD), and catalase (CAT) in the roots and leaves of waxy maize seedlings, reducing the production rate of superoxide anion (O_2_^-^) and the content of malondialdehyde (MDA) and proline (PRO) [29]. Studies have also shown that trehalose improves salt tolerance by increasing reactive oxygen species (ROS) scavenging capacity, alleviating plasma membrane damage, and maintaining cytoplasmic ion homeostasis [30,31]. Similarly, the application of exogenous trehalose promoted the growth of licorice seedlings and the accumulation of effective components under NaCl stress [32]. In wheat seedlings, exogenous trehalose improved the adaptation to salt stress by increasing proline accumulation and K^+^ absorption [33]. Additionally, Hu et al. showed that an appropriate concentration of trehalose simultaneously improved salt tolerance, drought tolerance, and cold tolerance of cucumber seedlings [34,35].

Watermelon (*Citrullus lanatus*) is an important economic horticultural crop worldwide. In 2019, watermelon ranked second and seventh in production and cultivated area among the world’s top ten fruits. Meanwhile, China is the world’s largest watermelon producing and consuming country, and the watermelon industry has played a significant role in increasing farmers’ income in China. However, soil salinization is a major problem affecting the production and quality of watermelon. According to survey statistics, about one billion hm^2^ of land worldwide is affected by salinization [36]. It is estimated that more than 50% of the global arable land area will be salinized by 2050 [37]. The area of salinized soil in China is thirty-six million hm^2^, accounting for 4.88% of the available land area, mainly distributed in the north and the coastal regions [38,39]. Studies have shown that most plants are damaged in soils with a salt content of up to 0.3% [40]. Salt stress mainly inhibits plant growth. However, with the aggravation of salt stress, the leaf area stops increasing, and the aboveground and underground fresh and dry weight decreases significantly [41]. Salt stress can either directly inhibit plant growth or indirectly affect plant growth by inhibiting photosynthesis and reducing the synthesis of growth substances. Moreover, the higher the salt concentration, the longer the action time, and the more noticeable the inhibition effect [42]. For watermelon, salt stress decreases planting area, yield, and quality [43]. Furthermore, with the annual expansion of the watermelon cultivation area, soil salinization will become more serious, which will have a serious impact on the sustainable and healthy development of watermelon.

Therefore, the present study investigated the functional classification, evolutionary characteristics, and expression profile of the *ClTPS* family. The study on the *ClTPS* gene will help breeders effectively select high-quality salt-tolerant germplasm resources to maintain watermelon production under adverse conditions. The study will also be of great significance for the transformation and utilization of saline-alkali land and the improvement of agricultural production levels.

## 2. Results

### 2.1. Identification of Watermelon TPS Genes and Distribution of TPS Proteins in Plant

Research has shown that TPS proteins contain two conserved domains, TPS and TPP [13,44]. Seven ClTPSs were identified from the watermelon genome database CuGenDB (http://cucurbitgenomics.org/organism/21) (accessed on 20 Octomber 2021) by BLASTP and numbered from ClTPS1 to ClTPS7 depending on their location on the chromosome (Table 1). The length of most of these TPS proteins (71.4%) ranged from 831 to 860 amino acids (aa), while the largest TPS (ClTPS2) had 933 aa and the smallest (ClTPS1) had 831 aa. The molecular weight (MW) ranged from 94.10 to 105.37 kDa and the predicted isoelectric points (pI) from 5.57 to 6.46. Finally, subcellular localization prediction indicated that the seven ClTPS proteins were located in the vacuole, including four showing chloroplast localization (ClTPS1, ClTPS4, ClTPS 6, and ClTPS7) and two showing cytoplasm localization (ClTPS2 and ClTPS3) too.

**Figure 1:**
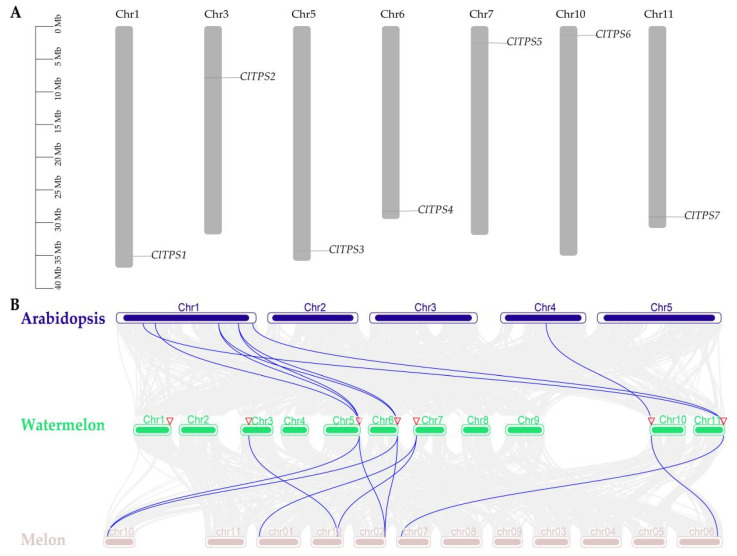
Chromosomal location of ClTPS genes and collinearity analysis of A. thaliana, watermelon and melon. (**A**) Chromosomal location of ClTPS genes; (**B**) collinearity analysis of A. thaliana, watermelon and melon.

Meanwhile, the seven ClTPSs were located in seven different chromosomes (chromosomes 1, 3, 5, 6, 7, 10, and 11) of watermelon (Figure 1A), and a gene duplication event was detected.

Furthermore, a collinear relationship diagram of the dicots watermelon, *A. thaliana*, and melon TPS family was constructed to further understand the evolutionary mechanism of the watermelon *TPS* family (Figure 1B). Eight pairs of direct homologous genes were identified between watermelon and *A. thaliana*, and nine between watermelon and melon. Collinearity analysis detected ClTPS3, ClTPS4, ClTPS6, and ClTPS7 in the three plants, suggesting that these genes may be highly conserved. In addition, the K_a_/K_s_ ratio of the eight pairs of direct homologous genes between watermelon and *A. thaliana* were less than 0.1 except for ClTPS7/AtTPS2 (supplementary Table S1), indicating purifying selection as the main driving force for the evolution of the watermelon *TPS* genes.

### 2.2. Phylogenetic Analysis, Structural and Conserved Motifs of ClTPSs

**Figure 2:**
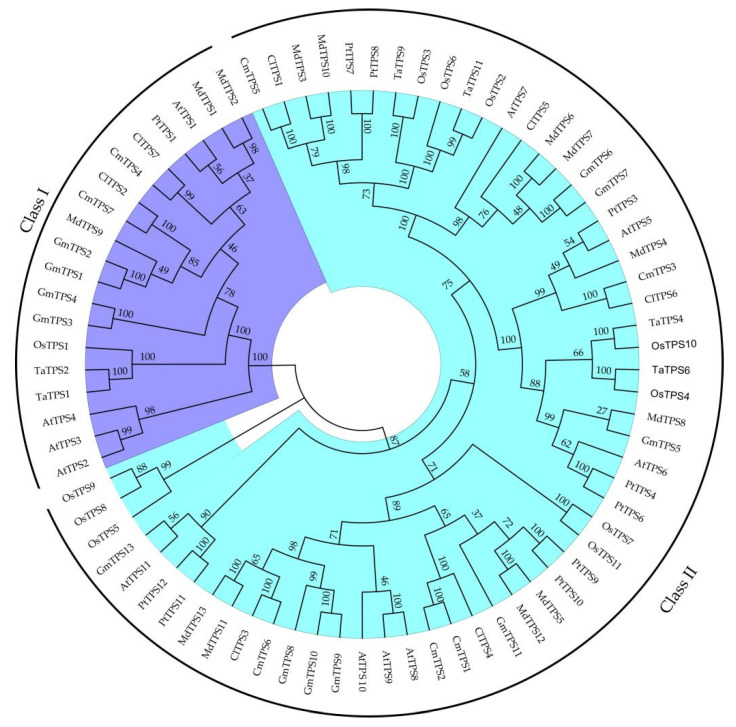
Phylogenetic relationship of TPS proteins among watermelon and other seven species. All TPS proteins were divided into two subgroups, represented by two colors. The green color represents Class I protein, and the blue color represents Class II protein. The phylogenetic tree was constructed by MEGA7 software with 1000 bootstrap replicates, following the neighbor-joining method.

**Figure 3:**
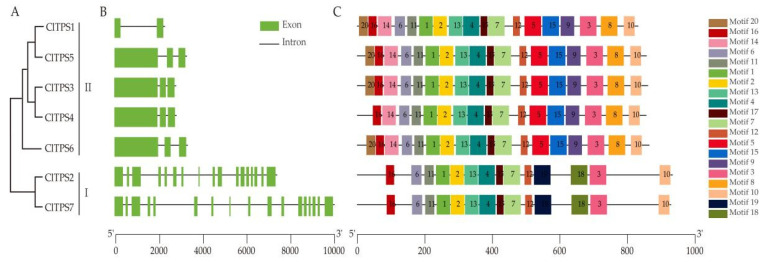
Phylogenetic analysis, gene structure, and conserved motifs of ClTPSs. (**A**) Phylogenetic analysis of seven ClTPSs. (**B**) Exon/intron organization of ClTPSs. (**C**) Conserved motifs of ClTPSs.

Seventy TPS proteins sequences from seven species and seven ClTPSs were used to construct a phylogenetic tree to understand the evolutionary relationship and classification of ClTPSs (Figure 2). All TPS proteins of watermelon were classified into two groups: Class I and Class II. Class I harbored two members, including ClTPS2 and ClTPS7; Class II harbored five members, including ClTPS1, ClTPS3, ClTPS4, ClTPS5, and ClTPS6. Phylogenetic analysis indicated that the protein was divided into two categories based on their sequences (Figure 3A). Generally, the most closely related members of each group had a similar exon–intron structure, with little difference in the length of introns and exons. Analysis of the exon–intron organization showed that all members except ClTPS1 in group II contain 3 exons, while group I members have 17 exons (Figure 3B).

Furthermore, MEME software was used to search for motifs and determine the predicted structural characteristics of ClTPS proteins (Figure 3C). Interestingly, all the members in group II contained 18 motifs, except ClTPS4 lacking motif 20; members of group I had only 14 motifs (lacking 5/8/9/14/15/20 motifs).

### 2.3. Prediction of Cis-Acting Elements of the ClTPS Genes

**Figure 4:**
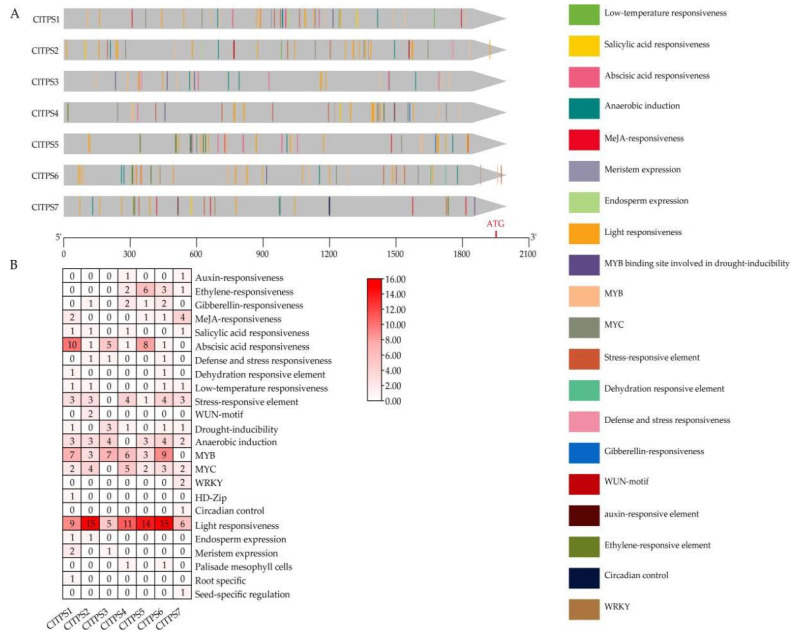
Cis-acting elements of ClTPSs. (**A**) Kind, quantity and position of cis-acting elements in ClTPSs; (**B**) numbers of cis-acting elements.

The 2000 bp sequences upstream of the start site of seven ClTPSs were used to identify the potential cis-acting elements in the promoter region for abiotic/biotic stress. The method predicted 249 cis-acting elements in these genes (Figure 4, Table S2). Many cis-acting elements were involved in response to environmental stress, hormone-responsiveness, development, light response, site binding, and other functions (Figure 4A). The most abundant elements were environmental stress-related elements, including those involved in low-temperature and anaerobic induction; all genes except CTPS7 contained low-temperature responsiveness elements, indicating that they may be considerably affected by the ambient temperature. Meanwhile, ABRE (ABA) was the most abundant among the predicted hormone-responsive elements (Figure 4B, Table S2).

### 2.4. Tissue-Specific Expression of ClTPS Genes

**Figure 5:**
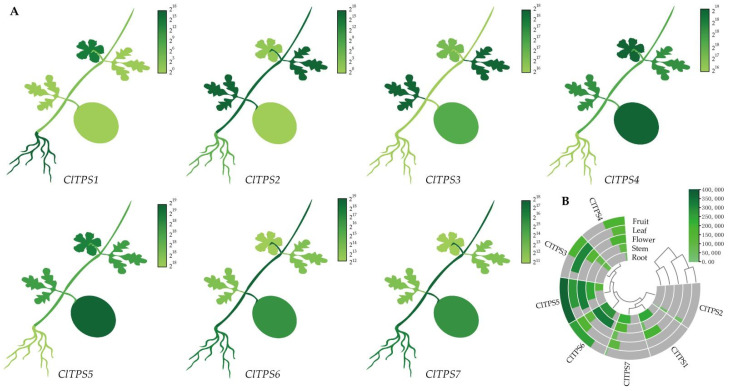
Tissue-specific expression of ClTPSs. (**A**) The heatmap shown in the form of the whole plant. (**B**) The heatmap and cluster analysis of tissue-specific expression. The expression levels were derived from NCBI and visualized by TBtools; the dark green represents high expression level, and the light green represents low expression level.

Initially, the transcript profiles derived from the NCBI database were used to analyze the expression levels of ClTPSs in watermelon flowers, fruits, stems, leaves, and roots to elucidate their functions and provide the basis for further understanding of the tissue-specific expression pattern (Figure 5). The seven ClTPSs were divided into two groups; the genes clustered in the same group had a similar expression pattern; for example, ClTPS1/2/7 had low expression levels in fruits and leaves, with no expression of ClTPS1 in fruits. The other ClTPSs were widely expressed in all tissues, with ClTPS5 and ClTPS6 showing relatively high expression levels throughout development.

### 2.5. Expression of ClTPS Genes under Different Salt Stress

**Figure 6:**
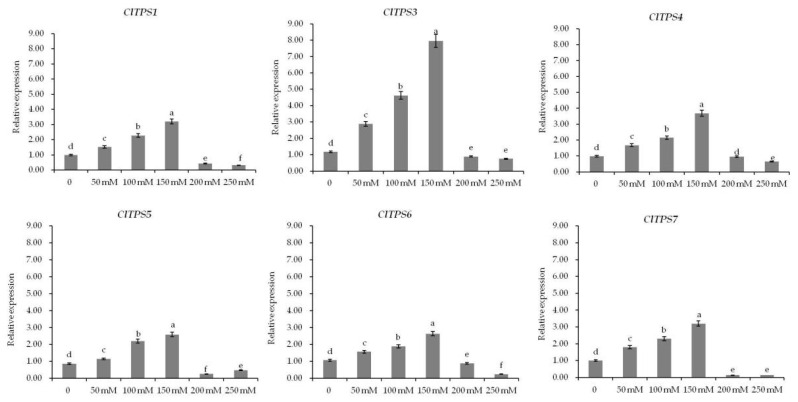
Expression analysis of ClTPSs under different NaCl concentrations. ClTPS2 expression was not detected. Error bars indicate the SD of three biological replicates. Different letters indicate significant differences within treatments by ANOVA (*p* < 0.05).

**Figure 7:**
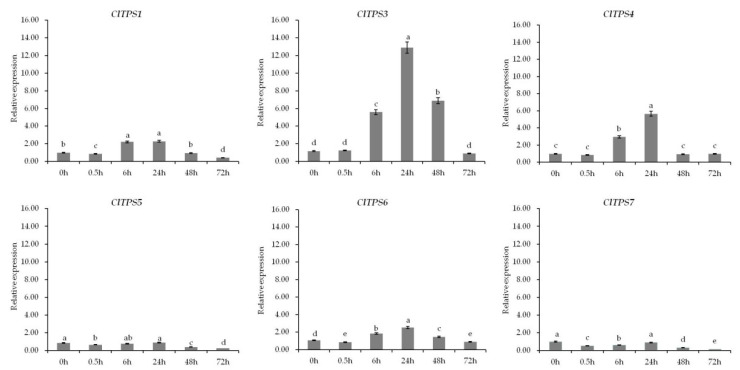
Expression analysis of ClTPSs at different time points under 200 mM NaCl stress. ClTPS2 expression was not detected. Error bars indicate the SD of three biological replicates. Different letters indicated significant differences within treatments by ANOVA (*p* < 0.05).

To further explore the role of *ClTPSs* in salt stress response, the expression levels of *ClTPSs* under different NaCl concentrations, including 0, 50, 100, 150, 200, and 250 mM, and at various time points, including 0, 0.5, 6, 24, 48, and 72 h, under 200 mM NaCl were analyzed by qRT-PCR results (Figure 6 and Figure 7). Under different NaCl concentrations, these genes (*ClTPS2* had no value) showed similar expression patterns (Figure 6). In the concentration range 0–150 mM, the expression levels of the six genes gradually increased and then decreased at 200–250 mM concentration range.

Further analysis of genes at different time points under 200 mM NaCl revealed that six genes showed a decrease towards 0.5 h, an increase from 0.5 to 24 h, and then a decrease from 24 to 72 h; the expression level peaked at 24 h (Figure 7). Dehydration, wilting, and death of watermelon seedlings under high osmotic conditions probably resulted in this expression pattern. Meanwhile, *ClTPS3* showed the highest expression level among all treatments, suggesting it is a key gene involved in salt stress response.

### 2.6. Functional Analysis of ClTPS3 Gene

The above results proved that salt stress significantly induced ClTPS3. A genetic transformation experiment was carried out in A. thaliana, and the plants were grown under 200 mM NaCl stress to verify the function of ClTPS3 further. The results showed that the expression level of *ClTPS3* in transgenic plants OE1, OE2, and OE3 was significantly higher than that of the wild-type plants (CK), which was 7.4, 7.8 and 8.2 times higher than CK, respectively (Figure 1).

**Figure 8:**
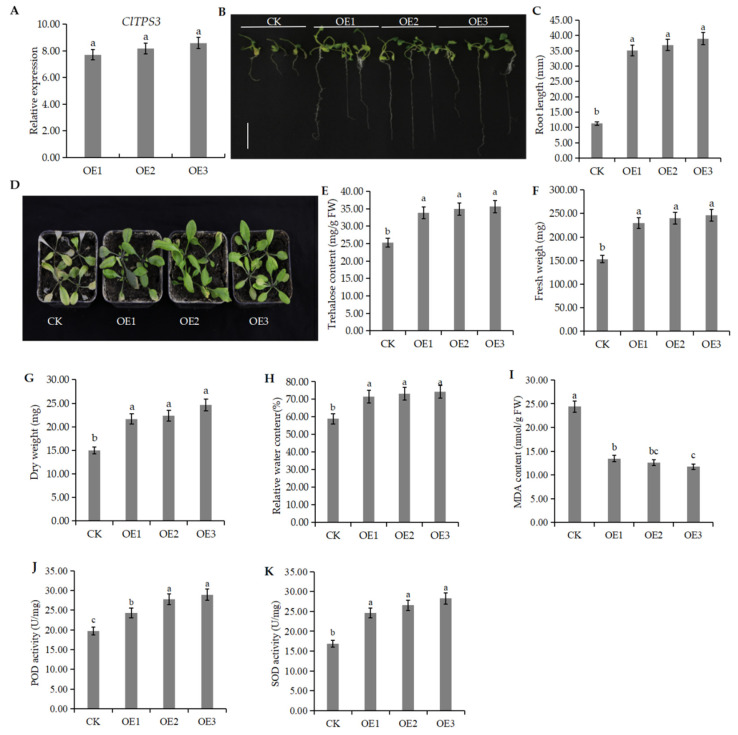
Phenotypic analysis of ClTPS3 overexpressed A.thaliana plants. (**A**) The expression level of ClTPS3 in different A.thaliana lines. (**B**) The growth status of wild type and transgenic seedlings under 200 mM NaCl stress, the white scale range represents 1 cm. (**C**) The root length of different A.thaliana lines under 200 mM NaCl stress. (**D**) The growth status of different A.thaliana lines adult plant under 200 mM NaCl stress. (**E**) Trehalose content of different A.thaliana lines. (**F**) Fresh weight, (**G**) dry weight, (**H**) relative water content, (**I**) MDA content, (**J**) POD activity and (**K**) SOD activity of different A.thaliana lines under 200 mM NaCl stress. CK represents wild-type A. thaliana plants, and OE1, OE2, and OE3 are transgenic A. thaliana plants. Error bars indicate the SD of three biological replicates. Different letters indicate significant differences within treatments by ANOVA (*p* < 0.05).

The transgenic plants grew normally under high salt concentration, while the growth of CK was significantly inhibited (Figure 8B). The transgenic plants had a root length of 35.19, 36.92, and 39.03 mm, respectively, which was three times more than that of CK (Figure 8C). To investigate the salt tolerance of transgenic plants and CK, the seedlings were treated with 200 mM NaCl for one week, and the results showed that transgenic plants had less wilting and a higher survival rate than CK (Figure 8C). To confirm this result, trehalose content, fresh weight (FW), dry weight (DW), and relative water content (RWC) were measured. The results showed that the trehalose content of transgenic plants was significantly higher than that of CK (Figure 8E), and the data of FW, DW and RWC exhibited similar trends (Figure 8F–H). In addition, the MDA content of the CK was significantly higher than that of transgenic plants, while POD activity and SOD activity were significantly lower than that of transgenic plants (Figure 8I–K), which indicates that ClTPS3-overexpressed reduced the content of H_2_O_2_ and O_2_− and reduced the cell membrane damage. These results suggest that the ClTPS3 gene may improve salt tolerance.

### 2.7. Functional Protein Association Networks of ClTPS Genes

**Figure 9:**
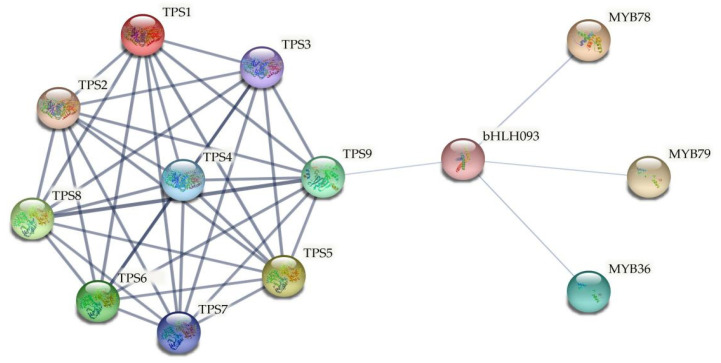
Functional protein association networks using the TAIR accessions of MYB36 (At5g57620.1, ClMYB9, Cla97C01G017110.1); MYB78 (At5g49620.2, ClMYB69, Cla97C09G167270.1); MYB79 (At4g13480.1, ClMYB51, Cla97C05G084530.1); bHLH093 (At5g65640.1, ClbHLH93, Cla97C11G220830.1); AtTPS1 (At1g78580.1, ClTPS1, Cla97C11G223240.1); AtTPS2 (At1g16980.1, ClTPS7, Cla97C11G223240.1); AtTPS3 (At1g17000.1, ClTPS7, Cla97C11G223240.1); AtTPS4 (At4g27550.1, ClTPS7, Cla97C11G223240.1); AtTPS5 (At4g17770.1, ClTPS6, Cla97C10G186050.1); AtTPS6 (At1g68020.1, ClTPS6, Cla97C10G186050.1); AtTPS7 (At1g06410.1, ClTPS5, Cla97C07G130930.1); AtTPS8 (At1g70290.1, ClTPS3, Cla97C05G107320.1); AtTPS9 (At1g23870.1, ClTPS3, Cla97C05G107320.1). Blue lines represent gene interaction confidence (0 to 1); thick lines indicate confidence score higher than 0.85, while thin lines indicate confidence score between 0.26 and 0.84.

As mentioned above (Figure 4), many MYB and bHLH transcription factor binding sites were detected in the promoter region of ClTPS genes, indicating their regulation by the transcription factors. A functional protein association network analysis was performed to screen for transcription factors that regulate ClTPS genes. Studies have reported the roles of MYB and bHLH transcription factor family genes in regulating watermelon response to abiotic stress [45,46]. Therefore, the interactions among the TPS proteins, MYB, and bHLH transcription factors were predicted using the STRING program. The analysis indicated a direct regulatory relationship between TPS9 (ClTPS3) and bHLH093 (ClbHLH93), while MYB36 (ClMYB9), MYB78 (ClMYB69), and MYB79 (ClMYB51) appeared to regulate bHLH093 (Figure 9).

## 3. Discussion

The present study adopted a bioinformatic approach to identify and characterize the watermelon *TPS* gene family. Seven *ClTPS* genes were identified in watermelon, which was less than the number of genes in *A. thaliana* and rice (11 *TPS* gene members). Previous studies revealed that fragment duplication resulted in three *TPS* genes in rice, genome-wide duplication produced one *TPS* gene in *A. thaliana*; *AtTPS2* and *AtTPS3* genes were produced by fragment duplication from *AtTPS1* [44]. However, in this study, no tandem duplication or fragment duplication event was detected in the *ClTPS* gene family, indicating different ancestor genes for these seven *ClTPSs*. This observation confirmed the presence of at least seven *TPS* genes in the common ancestor of monocotyledonous and dicotyledonous plants [47]. Meanwhile, in genetics, the K_a_/K_s_ ratio is used as an indicator of selective pressure (the force applied by natural selection) acting on a protein-coding gene. K_a_/K_s_ greater than 1 indicates a strong positive selection, genes with K_a_/K_s_ between 0.5 and 1 have a weak positive selection, and those with K_a_/K_s_ less than 1 are negatively selected (purifying selection) [48,49,50]. In this study, the analysis of evolutionary selective pressure indicated that the *ClTPS* genes were subjected to a purifying selection, which may partly explain fewer genes than *A. thaliana.*

According to the gene structure and enzyme activity, *TPS* family genes in plants are classified into two classes: Class I and Class II [51,52,53,54,55]. In the phylogenetic tree, *ClTPS2* and *ClTPS7* clustered with *AtTPS1*, *AtTPS2*, *AtTPS3*, *AtTPS4*, and *OsTPS1*, belonging to the Class I TPS subfamily, while the other five *ClTPS* genes belonged to the Class II TPS subfamily. Studies have also shown that the Class I genes have TPS activity, while the function of Class II TPS subfamily genes is not clear [52,56,57,58]. However, few researchers pointed out that the N-terminal of some genes in Class II TPS subfamily have the TPS domain of the conserved sites of glycotransferase and the TPP domain of the conserved sites of two phosphohydrolases at the C-terminal; the presence of these two domains indicate that the gene members are either bifunctional or act as a TPS complex subunit [54,55,59]. Long et al. [28] predicted ten glycosylation sites and forty-nine phosphorylation sites for the protein encoded by the *ClTPS1* gene, indicating TPS and TPP activities for the *ClTPS1* gene. The functional verification of the *TPS* gene family in maize revealed both TPS activity and TPP activity for the *ZmTPS3* gene of the Class II subfamily [60]. These observations collectively indicate that the TPS genes may quickly respond to stress and synthesize trehalose to help watermelon survive under stress.

The *ClTPS* genes of Class I and Class II subfamily had significantly different numbers of exons, indicating evolutionary differences between them similar to *Populus*, *A. thaliana*, and rice [44]. Studies have proven a close relationship between the structure and function of genes [61]. Similarly, the differences in the expression patterns and functions of Class I and Class II *ClTPS* genes may be related to the wide differences in the exon–intron structure. In this study, two Class I members and four Class II members showed similar tissue expression patterns (Figure 5B), probably because few introns are required during the selective splicing of mRNA to subsequently regulate the structure and function of the protein encoded by the gene. Meanwhile, some genes may have enhanced mRNA transcription and transport, leading to different tissue-specific patterns and different expression levels [62,63].

*TPS* is a gene that encodes a key enzyme in the trehalose pathway. The promoter region of *ClTPS* genes contains a variety of signal response elements, which can respond to various stress conditions. Moreover, each *ClTPS* gene contains different stress response elements, indicating that different signals may induce each gene. The *ClTPS5* gene has the largest number of hormone-responsive *cis*-acting elements, consistent with the highest expression level in the tissues. Many *cis*-acting elements involved in drought-inducibility and anaerobic induction were identified in the *ClTPS3* gene, in agreement with the high expression level under osmotic stress. Studies have demonstrated that an important feature of drought and salt stress is cell osmosis, which leads to ABA accumulation as an adaptive response [64]. Meanwhile, the relationship between *ScTPS* and ABA signals in sugarcane indicated the role of *ScTPS* in drought tolerance [65]. Most of the *ClTPS* genes also had ABA *cis*-acting elements, suggesting their roles in the drought stress response.

Studies have shown that high temperature, high salt concentration, and drought stress significantly increased the expression levels of *TPS1* and *TPS7* genes in potatoes, suggesting the role of the *TPS* gene in the signal transduction pathway of stress tolerance [13]. Similarly, the expression level of *PhTPS6* in petunia was significantly upregulated after 12 h of low-temperature treatment, with a gradual increase in the expression level with the treatment time [66]. *PhTPS6* showed the same expression pattern after NaCl treatment, suggesting its role under low temperature and salt stress [66]. A recent study in sugarcane showed that salt and drought induced the expression of the *ScTPS1* gene, which indicated that sugarcane maintained the cell osmotic stability by increasing trehalose-6-phosphate production and alleviated the simulated stress. These observations suggested that genetic engineering of endogenous *ScTPS1* gene involved in trehalose biosynthesis may improve the drought tolerance of sugarcane [13]. Similarly, salt stress markedly induced *ClTPS3* (Figure 6 and Figure 7). Previous studies had proved that trehalose could improve the salt tolerance of plants by increasing the activity of antioxidant enzymes, and it was found that the activities of SOD, CAT, POD were positively correlated with the salt tolerance of plants [29,67]. In addition, trehalose significantly reduced the accumulation of MDA in tomato, indicating that trehalose can reduce the degree of cell membrane damage and improve the salt tolerance of tomato [68]. In this study, the trehalose content of *ClTPS3* transgenic *A.thaliana* plants was significantly increased, and the activities of SOD and POD were also significantly increased. In addition, the MDA content was significantly decreased, while the relative water content was significantly increased, indicating that *ClTPS3* played an important role in the salt tolerance of watermelon. Furthermore, the results of STRING functional protein association networks showed that ClMYB and ClbHLH transcription factors most likely regulated only *ClTPS3* among the seven *ClTPS* genes. These results indicate a putative role of *ClTPS3* in watermelon response to various abiotic stresses. Therefore, effective use of the *ClTPS3* gene may help watermelon maintain growth and production under salt stress.

## 4. Materials and Methods

### 4.1. Identification and Characterization Analysis of Putative Watermelon TPS Genes

In this study, 11 AtTPS protein sequences and 11 OsTPS protein sequences [44] were used to blast against the CuGenDB (http://cucurbitgenomics.org/organism/21) (accessed on 20 Octomber 2021) [69] to identify TPS genes of watermelon. The Conserved Domains database (https://www.ncbi.nlm.nih.gov/Structure/cdd/wrpsb.cgi) (accessed on 8 June 2021) was used to ensure that all candidate TPSs contained the TPS domain. In addition, the online software ExPASy Proteomics Server (http://web.expasy.org/protparam/) (accessed on 8 June 2021) was used to analyze the molecular weight, length, and isoelectric point of the watermelon TPS proteins [70], and the online software Cell-PLoc (http://www.csbio.sjtu.edu.cn/bioinf/Cell-PLoc-2/) (accessed on 8 June 2021) was used to predict their subcellular localization [71].

### 4.2. Chromosomal Localization, Phylogenetic Analysis, Duplication Events, and Collinearity Analysis of ClTPS Genes

The chromosomal distribution of ClTPSs was derived from CuGenDB. The TBtools software [72] was used to determine the chromosome localization and the ClTPSs duplication events. Furthermore, 77 protein sequences of various species, including watermelon, Populous, Cucumis melo, Malus domestica, A. thaliana, Oryza sativa, Glycine max, and Triticum aestivum, were used to construct the phylogenetic tree. Amino acid sequence alignment was carried out using Clustal W [73], and then the phylogenetic trees were constructed following the neighbor-joining (NJ) method with 1000 bootstrap using the MEGA 7.0 software [74]. TPS genes duplication events, collinearity, and selective evolutionary pressure were analyzed using the TBtools software [72].

### 4.3. Analysis of ClTPS Gene Structures and Conserved Motifs

The structure (exon-intron arrangement) of ClTPS genes was obtained from CuGenDB and visualized using TBtools. Then, the putative conserved motifs of ClTPS protein sequences were analyzed using the MEME program (http://meme-suite.org/tools/meme) (accessed on 8 June 2021), with any number of repetitions, a maximum of 20 motifs; and an optimum width of each motif between 6 and 60 residues [75]; the conserved domains were visualized using TBtools.

### 4.4. Analysis of Cis-Acting Elements

TBtools was used to extract the 2000 bp sequences upstream of the transcriptional start site (ATG) of the ClTPS genes as the putative promoter region; these sequences were submitted to Plantcare (http://bioinformatics.psb.ugent.be/webtools/plantcare/html/) (accessed on 8 June 2021) to identify the cis-acting elements in the promoter region.

### 4.5. Tissue-Specific Expression Patterns and Expression Levels under Different Stresses of ClTPS Genes

The data of different tissues, including flower (GSE69073), fruit (PRJNA338036), stem (SRP012853), leaf (PRJNA381300), and root (PRJNA641525), and under different stresses, such as drought (GSE144814), osmotic stress (PRJNA381300), heat shock (PRJNA504354) and cold (PRJNA328189), were retrieved from the NCBI database (https://www.ncbi.nlm.nih.gov/) (accessed on 22 June 2021). The data were first downloaded from the SRA database, converted into fastq format, and uploaded into the Kallisto Super Wrapper of TBtools to obtain the transcript expression matrix. Finally, the gene expression matrix was obtained from the transcript expression matrix using the Trans Value Sum of TBtools. Furthermore, hierarchical clustering and heatmap of ClTPS genes were generated using TBtools.

### 4.6. Plant Growth and Treatments

Watermelon (“HQ-2” variety) seedlings were maintained in Hoagland solution in a growth chamber at 25 °C under a photoperiod of 16 h light/8 h dark. After growing for 30 days, the seedlings were grown hydroponically in Hoagland solution containing 0 (control), 50, 100, 150, 200, or 250 mM NaCl; thirty seedlings were maintained per treatment. Control plants (were cultured in standard Hoagland solution) were cultured in parallel. Seedlings were sampled after 3 days of salt treatment. In addition, samples under 200 mM NaCl were collected at 0, 0.5, 6, 24, 48, and 72 h. Finally, all the samples were immediately frozen in liquid nitrogen and stored at −80 °C until for RNA extraction. Three seedlings were mixed into one sample, and three biological replicates were maintained per treatment.

### 4.7. RNA Extraction and Quantitative Real-Time-PCR

Total RNA was isolated using the Plant RNA Kit (Huayueyang, Beijing, China) according to the manufacturer’s protocol. The first strand of cDNA was obtained using a PrimeScript RT reagent Kit (TaKaRa, Dalian, China). qRT-PCR was performed on the Light Cycler480 Real-Time System (Bio-Rad Laboratories) with the method described previously [61]. *ClActin* was used as the reference gene, and the specific primers are shown in Supplementary Table S3. The data were analyzed using the 2^−ΔΔCt^ method [76].

### 4.8. Functional Analysis of ClTPS3 Gene

The coding sequence of *ClTPS3* (Cla97C05G107320.1) was inserted into the plant transformation vector pRI101-AN at Nde I-Kpn I sites to generate the *ClTPS3*-overexpressing recombinant vector, and the specific primer sequences used for PCR amplification were as follows: 5′-CATATGATGGCATCAAGATCCCCCAC-3′ and 5′-GGTACCTCAAAAAACACTCTCAAAAGAAACAC-3′. The construct was transformed into *Agrobacterium tumefaciens* strain GV3101.

The *Agrobacterium* dipping flower method was employed to infect *A. thaliana* [77]. The seeds of the overexpressed homozygote and the wild-type *A. thaliana* were disinfected and seeded on the MS medium and cultured under normal light. The overexpressed (*OE*) and wild-type *A.thaliana* (CK) plants were transferred to an MS medium containing 200 mM NaCl for stress treatment. After 7 days, the root length was measured using a vernier caliper (SHRN 0–300 mm, Guilin, China), and at least ten seedlings were measured for each line. In addition, the 3-week-old *OE* and CK seedlings were treated with 200 mM NaCl for 7 days, then three seedlings were sampled for the fresh weight (FW), dry weight (DW) and relative water content (RWC) measurement, and ten seedlings were immediately frozen in liquid nitrogen and stored at −80 °C until for RNA extraction and indexes analysis.

Furthermore, the trehalose, MDA, POD, and SOD content were determined using BC0330 (Solarbio, Beijing, China), KTB1050, KTB1150, and KTB1030 (Abbkine, Beijing, China) kits, respectively. The absorbance was measured using the SpectraMax i3X Multi-mode Detection Platform Molecular Devices (Molecular Devices, China).

All data were statistically analyzed using three biological replicates and expressed as mean values ± standard deviation (SD). SPSS 18.0 statistical software was used for variance analysis, and differences were considered statistically significant at a *p*-value of 0.05 (*p* < 0.05).

### 4.9. Functional Protein Association Networks of ClTPS Genes

A TPS-related functional protein association network was built using the online software STRING (http://stringdb.org) (accessed on 1 May 2021) using the TAIR gene ID of genes related to *TPS*. The amino acid sequences of the MYB transcription factors and bHLH transcription factors were derived from the articles of Wang [45] and He [46]. The gene ID of *A. thaliana* may be responsible for multiple members in watermelon. The transcription factors with the highest amino acid identity were selected (Table S4). The confidence level of minimum required interaction score parameters was set at 0.25.

## 5. Conclusions

In this study, a total of seven *TPS* genes were identified through genome-wide analysis in watermelon, divided into two groups, and located on seven chromosomes. Tissue-specific expression and stress response analysis showed diversity and specificity in the expression patterns of *ClTPSs* in different tissues and under various stresses. The *ClTPS3* gene was highly expressed under salt stress, suggesting an essential role in watermelon’s response to salt stress. Our results will help to lay a foundation for further understanding the structures and characteristics of the *TPS* gene family and improving the efficiency of watermelon breeding.

## Figures and Tables

**Table 1:** Characteristics of *TPS* family members in watermelon.

Gene Name	Gene ID	Length ORF (bp)	No. of Amino Acids	TPS Domain Location	TPP Domain Location	Isoelectronic Point (*pI*)	Molecular Mass (kD)	Subcellular Localization
ClTPS1	Cla97C01G023850.1	2496	831	41–525	574–808	5.57	94.10	Chloroplast/Vacuole
ClTPS2	Cla97C03G058540.1	2802	933	92–559	593–817	6.46	105.37	Cytoplasm/Vacuole
ClTPS3	Cla97C05G107320.1	2583	860	58–545	594–828	6.15	97.32	Cytoplasm/Vacuole
ClTPS4	Cla97C06G126510.1	2568	855	53–540	589–823	6.38	97.23	Chloroplast/Vacuole
ClTPS5	Cla97C07G130930.1	2571	856	60–544	593–827	5.66	97.47	Vacuole
ClTPS6	Cla97C10G186050.1	2595	864	63–548	597–829	5.81	97.54	Chloroplast/Vacuole
ClTPS7	Cla97C11G223240.1	2787	928	94–561	595–816	6.39	105.10	Chloroplast/Vacuole

## Data Availability

Not applicable.

## Supplementary Materials

The following are available online at https://www.mdpi.com/article/10.3390/ijms23010276/s1.

## Abbreviations

T6P	trehalose-6-phosphate
UDPG	uridine diphosphate glucose
TPS	trehalose-6-phosphate synthetase
TPP	trehalose-6-phosphate phosphatase
TRE	trehalase
SOD	superoxide dismutase
APX	ascorbic acid peroxidase
POD	peroxidase
CAT	catalase
MDA	malondialdehyde
O2-	superoxide anion
PRO	proline
ROS	reactive oxygen species
ORF	open reading frame
pI	isoelectronic point
MWs	molecular weights
NJ	neighbor-joining
FW	fresh weight
DW	dry weight
RWC	relative water content
